# Association of Indocyanine Green with Chitosan Oleate Coated PLGA Nanoparticles for Photodynamic Therapy

**DOI:** 10.3390/pharmaceutics14081740

**Published:** 2022-08-20

**Authors:** Dalila Miele, Milena Sorrenti, Laura Catenacci, Paolo Minzioni, Giorgio Marrubini, Valeria Amendola, Marcello Maestri, Paolo Giunchedi, Maria Cristina Bonferoni

**Affiliations:** 1 Department of Drug Sciences, University of Pavia, 27100 Pavia, Italy; 2 Department of Electrical, Computer and Biomedical Engineering, University of Pavia, 27100 Pavia, Italy; 3 Department of Chemistry, University of Pavia, 27100 Pavia, Italy; 4 IRCCS Policlinico San Matteo Foundation, 27100 Pavia, Italy; 5 Department of Medicine, Surgery and Pharmacy, University of Sassari, 07100 Sassari, Italy

**Keywords:** indocyanine green, photodynamic effect, chitosan oleate, PLGA nanoparticles

## Abstract

Indocyanine green (ICG) is a safe dye widely used in the biomedical field. Its photodynamic effect (PDT), originating from laser irradiation at 803 nm, opens interesting perspectives in theranostic applications. To overcome its low water stability, ICG can be shielded with nanoparticles (NPs). In this work, previously developed NPs based on poly lactic-co-glycolic acid (PLGA) coated with chitosan oleate (CS-OA) and loaded with resveratrol as a hydrophobic model drug have been proposed as an ICG carrier. These systems have been selected for their observed immunostimulatory properties. The possible loading of the dye by adsorption onto NP surface by electrostatic interaction was studied here in comparison with the encapsulation into the PLGA core. The ICG-chitosan (CS) interaction has been characterized by spectrophotometry, spectroscopy and in-cell in vitro assays. Fluorescence quenching was observed due to the ionic interaction between ICG and CS and was studied considering the dye:polymer stoichiometry and the effect of the NP dilution in cell culture medium (DMEM). The NP systems have been compared in vitro, assessing their behaviour in Caco-2 cell lines. A reduction in cell viability was observed after irradiation of ICG associated with NPs, evident also for the samples loaded by adsorption. These findings open the opportunity to exploit the association of PDT’s effect on ICG with the properties of CS-OA coated NPs, whose immunostimulatory effect can be associated with PDT mechanism in cancer therapy.

## 1. Introduction

Indocyanine green (ICG) is a water-soluble amphiphilic cyanine derivative with fluorescence properties in the near-infrared region (NIR). It absorbs at 800–810 nm and emits at slighter longer wavelengths, 830 nm [1,2]. This molecule is widely used as an imaging dye in the biomedical field. Thanks to the low toxicity indicated by LD50 of 50–80 mg/kg after intravenous administration [3], ICG has been approved for clinical use by the European Medicines Agency (EMA) and the U.S. Food and Drug Administration (FDA) for intravascular, interstitial and eye imaging applications [4]. In detail, ICG is used in the oncology area for the intraoperative identification of solid tumors and metastases in the liver and in colorectal surgery as it improves the localization of tumor margins with a definition of a few millimeters, ameliorating the success of the total tumor resection [5,6,7,8]. Its efficiency as a tissue penetrator and its minimal auto-fluorescence after its interaction with biological substrates are the major benefits of ICG [9]. In addition, this dye can provide the detection of sentinel lymph nodes by optical imaging [10]. 

ICG is also widely applied in theranostics for tumor diagnosis and treatment [1]. It can be used as an agent for the eradication of malignant tissue through two distinct approaches: photothermal therapy (PTT) and photodynamic therapy (PDT). In the first procedure, the tumor mass is reduced by the induction of a hyperthermic effect activated by irradiation, while PDT involves the production of reactive oxygen species [11,12]. 

Nevertheless, some drawbacks can limit its clinical usage, such as the occurrence of degradation and aggregation in aqueous solutions [13]. ICG distribution after systemic administration is limited due to its quick hepatobiliary elimination and the consequent rapid blood clearance, which restricts its accumulation in the tumors [14]. For all these reasons, the loading of the dye into different carriers has been proposed to improve its stability, prolong its blood circulation and target it specifically to the tumors through passive and/or active pathways, therefore reducing the toxicity [15,16,17].

A large number of nanosystems have been proposed in the literature [17], such as inorganic [18], polymeric [19,20] and lipid nanoparticles [21], as well as liposomes [22]. Moreover, the loading of a photosensitizer into nanocarriers can improve the PDT efficiency by preventing photosensitizer aggregation [17,23]. 

Chitosan (CS) is a linear polycationic amino-polysaccharide (poly-1,4-D-glucosamine), whose amino groups are ionized in a weakly acidic environment, determining the possibility of interaction of the polymer with negatively charged surfaces, e.g., cell membranes [24,25]. Besides the capability of electrostatic interactions, CS has been reported to be able to open tight junctions, and therefore, it is widely utilized for the development of pharmaceutical formulations administered through different routes, including oral, ophthalmic, nasal, and transmucosal routes [26,27,28]. It has also been proposed in formulations for hepatocellular carcinoma (HCC) treatment [29,30]. Some examples can be found in the literature of nanoparticles (NPs) as ICG carriers for PDT or PTT and based on CS, in many cases combined with different materials in hybrid structures. Jheng et al. proposed a CS-arginine derivative to improve the stability of ICG NPs loaded with doxorubicin [31]. PLGA NPs coated with zwitterionic CS have been studied by Park et al. [32]. Chen et al. encapsulated ICG in gold nanorods (AuNR)-CS hybrid nanospheres (CS-AuNR-ICG NSs) [33]. Quite recently, Lee et al. coated liposomes with CS to improve ICG’s transdermal efficacy in the treatment of melanoma [34], and Liu et al. designed a heat-sensitive ICG delivery system based on carboxymethyl CS [35]. In the systems developed by Sanchez-Ramirez et al., the presence of CS improved the encapsulation of ICG in carboplatin-loaded PLGA NPs [36].

In our previous works, the amphiphilic properties of a hydrophobically modified CS obtained by electrostatic interaction between CS amino groups and oleic acid (OA) carboxylic groups have been demonstrated to be useful for easily preparing PLGA NPs coated with a chitosan oleate (CS-OA) shell [37]. The positive charge of the NPs was successfully exploited to load a negatively charged siRNA [38]. In the same work, the CS-OA immunostimulatory properties were put into evidence. These properties make the system worthy of being studied when combined with ICG. Even in this case, the ionic interaction between positive NPs and the negative charge of ICG can be exploited. This approach involves immediate NP-ICG interaction and can be easily applied to NPs previously loaded with different actives, such as resveratrol (RSV), without concern about the compatibility between ICG and the NP cargo. The immunomodulatory properties of the CS-OA NPs [38] can also be useful for their application in cancer therapy, as suggested by some literature evidence. Kumar et al., in fact, proposed the exploitation of the immunostimulatory properties of glycol CS associated with polycaprolactone and poloxamer for the treatment of cancer by photoimmunotherapy [39]. Considering that tumors subject to photothermal ablation generated tumor-associated antigens, Chen et al. developed an NP system loaded with ICG and enriched with an immunostimulant drug such as imiquimod [40].

From the perspective of developing NPs suitable for PDT of cancer, the aim of the present study was the characterization of the electrostatic interaction between CS-OA-coated PLGA NPs and adsorbed ICG, in comparison with NPs with ICG encapsulated in a PLGA core. In particular, the relevance of ICG and NP interaction for the fluorescence and for the effect on Caco-2 cell culture treatment was investigated. Previously developed PLGA-NPs loaded with RSV, here used as a model hydrophobic molecule, were in the present work selected for ICG loading [41].

## 2. Materials and Methods

### 2.1. Materials

Chitosan LMW (CS) (80% deacetylation degree, DD), poly-lactic-glycolic acid (PLGA) (Resomer RG 503H), and Nile red were purchased from Sigma-Aldrich (Milan, Italy). Oleic acid (OA) was purchased from Fluka (Milan, Italy), and 99% pure trans-resveratrol (RSV) was purchased from Mega Resveratrol (Candlewood Stars Inc., Danbury, CT, USA). Acetone, ethyl acetate, acetic acid, sodium acetate and methanol were acquired from Carlo Erba (Milan, Italy).

### 2.2. Preparation of Nanoparticles

CS was added to 100 mL of bidistilled water under magnetic stirring (300 rpm) containing 250 µL of glacial acetic acid to obtain a 1% *w*/*w* polymer concentration. The functionalization of 50% of CS binding sites with OA was achieved by solubilizing a stoichiometric amount of fatty acid in acetone, added dropwise to the CS solution. A chitosan oleate (CS-OA) salt was obtained after acetone evaporation; CS-OA was freeze-dried and used to obtain PLGA-NPs according to the solvent evaporation method previously described [37]. Briefly, 12 mg of CS-OA was dispersed in 5 mL of distilled water saturated with ethyl acetate, and 6 mg of PLGA in 0.25 mL of ethyl acetate was added and emulsified at 20,500 rpm by means of Ultra-Turrax T25 (Janke & Kunkel, IKA Labortechnik, Germany) equipped with an 8 mm probe (S25 N-8 G). After 5 min, 5 mL of distilled water was added, and emulsification was carried out for a further 5 min. Ethyl acetate was removed under stirring overnight. NPs were sonicated for 15 min in an ice bath and centrifuged for 10 min at 3000 rpm. To obtain RSV-loaded PLGA-NPs, RSV was added to the organic phase together with PLGA, in a final concentration of 0.5 mg/mL. RSV-loaded NPs were prepared in amber glass vials. An adsorption method was used for the functionalization with ICG of both unloaded and RSV-loaded NPs. Different volumes of a 0.4 mg/mL ICG aqueous solution were dropped under stirring in a diluted suspension of NPs (0.24 mg/mL CS-OA) to obtain ICG:CS molar ratios ranging from 0.02:1 to 0.4:1, considering CS monomeric units. For confocal laser scanning microscopy (CLSM) visualization, NPs loaded with Nile red were obtained by adding Nile red (5 µg/mL) instead of RSV and were functionalized by adsorption with ICG at an ICG:CS molar ratio of 0.4. The RSV-loaded NPs were functionalized with ICG by the encapsulation method as well. In this method, an amount of ICG corresponding to a 0.4 ICG:CS molar ratio was added to the PLGA in ethyl acetate during the emulsification step. 

### 2.3. Particle Size and Zeta Potential Characterization

The mean particle size and the polydispersity index (PI) of the samples were assessed by means of photon correlation spectroscopy (PCS; N5 submicron particle size analyzer, Beckman Coulter, Milan, Italy). Analyses were carried out at a 90° detection angle by diluting the sample in distilled and filtered (0.22 µm) water according to the apparatus instructions. Zeta potential was evaluated by a Zetasizer nano series (Malvern Instruments Ltd., Worcestershire, UK) in aqueous suspension.

### 2.4. ATR Fourier-Transform Infrared (FT-IR) Spectroscopy

FT-IR spectra were performed on commercial ICG and on CS-OA prepared according to the method previously described and then freeze-dried. The interaction product between CS-OA and ICG was prepared by dispersing CS-OA in distilled water and by adding an amount of ICG corresponding to an ICG:CS molar ratio of 0.2:1. A solid sample was obtained by freeze-drying. FT-IR spectra were recorded using a Fourier transform infrared spectrophotometer (Perkin Elmer SpectrumOne, Milan, Italy) with a single reflection ATR accessory (PIKE MIRacle). Approximately 10 mg of each sample as such were placed on ATR crystal of ZnSe and pressed down to the crystal. The spectra were collected in transmittance mode, at least in triplicate, with a resolution of 4 cm^−1^ within the spectral range of 650–4000 cm^−1^ [41,42].

### 2.5. UV-Visible and Fluorescence Spectroscopy

The UV-visible spectra were acquired in distilled water in a wavelength range between 350 and 900 nm by means of a Perkin Elmer Lambda 25 spectrophotometer (Perkin Elmer, Milan, Italy). The spectra were recorded immediately after the preparation (time 0) and repeated after 1 or 3 weeks of storage at 4 °C.

Spectrofluorimetric spectra were collected by means of a Perkin Elmer LS50B apparatus (Perkin Elmer, Milan, Italy) in an emission range between 730 and 880 nm with excitation at 709 nm and slits at 15 nm. Fluorescence intensity was expressed in arbitrary units (a.u.). ICG spectra were registered at the zero timepoint and after 2 weeks of storage at 4 °C for aqueous solutions and NP samples functionalized with ICG either for adsorption (ads-ICG NPs) or for encapsulation (enc-ICG NPs), as described in Section 2.2.

ICG spectra were also registered after mixing ICG with increasing concentrations of CS both associated with the NPs and free. ICG:CS molar ratios ranging from 8:1 to 0.4:1 were tested. The spectra were acquired both in aqueous solutions and in cell culture medium, DMEM (Dulbecco’s modified Eagle’s medium, Sigma Aldrich, Milan, Italy), with the addition of 1% (*v*/*v*) penicillin-streptomycin-amphotericin 100X (Biowest, Riverside, MO, USA), 10% *v*/*v* inactivated fetal bovine serum (Biowest, Riverside, MO, USA) and 1% *v*/*v* non-essential aminoacid solution 100X (Sigma Aldrich, Milan, Italy).

### 2.6. Sephadex G-25 Separation of ICG Free and Associated to the NPs

To separate free ICG and NPs, a gel filtration procedure was developed by modifying literature methods [43,44]. Here, 2 g Sephadex G-25 (Fine, Sigma Aldrich, Milan, Italy) was hydrated in distilled water, and a chromatographic column was packed by gravity. A flux of 0.4 mL/min was maintained by means of an HPLC pump (isocratic Kontron 420, Kontron Instruments, Milan, Italy). Then, 250 µL of ICG aqueous solution 0.4 M was loaded as reference. Subsequently, 1 mL fractions were collected and analyzed at 780 nm wavelength by means of a spectrophotometer (Perkin Elmer Lambda 25, Milan, Italy) versus a calibration curve. Different amounts of NPs were mixed with the same amount of ICG solution of the reference experiment and loaded in the column, obtaining molar ratios between ICG and CS (as a monomeric unit) ranging from 8:1 to 0.4:1. In these cases, as well, 1 mL volumes were collected and analyzed for ICG concentration. A similar procedure was followed when loading ads-ICG NPs and enc-ICG NPs, both at an ICG:CS molar ratio of 0.4. These were, however, eluted not only with distilled water but also with DMEM cell culture medium supplemented with 10% *v*/*v* inactivated fetal calf bovine serum (Euroclone, Milan, Italy).

### 2.7. Cell Culture Test

#### 2.7.1. Caco-2 Cell Culture

Caco-2 cells (Sigma Aldrich, Milan, Italy) were cultured in DMEM supplemented with 1% *v*/*v* antibiotic/antimycotic solution (Euroclone, Milan, Italy) and 10% *v*/*v* inactivated fetal calf bovine serum (Euroclone, Milan, Italy); 1% *v*/*v* of non-essential amino acid (Mem Non—Essential Amino Acid Solution 100X; Sigma Aldrich, Milan, Italy) was added to the medium. Cells were seeded in 48-well plates (5 × 10^4^ cells/well) and incubated (37 °C and 5% CO_2_ atmosphere) for 24 h to reach semi-confluence. 

#### 2.7.2. Confocal Laser Scanning Microscopy (CLSM) 

The visualization of NP internalization in cell substrates was assessed by confocal microscopy [35,41]. CLSM analyses were conducted as described below: a microscope slide (Ø = 13 mm) was inserted into each well in a 24-well plate, and 10 × 10^5^ cells were seeded in 500 µL of medium. After 24 h culture, cells were kept in contact with the Nile red loaded sample and diluted in medium at the same concentration used for laser irradiation test, for further 24 h. Afterwards, cells were washed twice with 500 µL of phosphate-buffered solution pH 7.4 (PBS) and fixed for 15 min at 4 °C with a 4% (*v*/*v*) paraformaldehyde solution diluted in PBS. Then, substrates were flushed with 500 µL of PBS and, before confocal microscopy analysis, nuclei were stained with 100 µL of Hoechst 33258 (diluted 1:100,000 in PBS) for 15 min. Finally, the Hoechst was removed, and cells were washed with PBS. The slides with fixed cells were removed from the wells and were examined with CLSM (Leica TCS SP5II, GmbH) by setting the fluorescence of labeled nuclei (Hoechst 33258, λ_ex_ = 346 nm and λ_em_ = 460 nm) and Nile red (Nile red, λ_ex_ = 440 nm and λ_em_ = 520 nm).

#### 2.7.3. Sample Loading and Laser Irradiation

All samples were diluted with medium to achieve a non-cytotoxic ICG concentration of 13 µM. All the samples were kept in contact with the cells for 24 h; then, in half of the wells, the ICG was removed and replaced with fresh medium, while in the other half, cells were kept in contact with the cyanine derivative. 

Each well was irradiated with a NIR laser beam, emitted by an AlGaAs semiconductor source (λ = 808 nm) [31]. The laser source was thermally controlled and kept at 21 °C, while a cylindrical lens with a focal length of 60 mm was introduced to reduce the strong astigmatism of the beam (θ_x_ = 12°; θ_y_ = 40°). An iris was used to select the central portion of the beam, thus guaranteeing to irradiate an area corresponding to one well only. The homogeneity of the irradiation was characterized by using a CCD, and the analysis yielded a variation coefficient of 0.3. Irradiation was carried out by delivering a dose of 90 J/cm^2^ using an intensity of either 100 mW/cm^2^ or 300 mW/cm^2^ and an irradiation time of 15 or 5 min, respectively. 

#### 2.7.4. Cell Viability Test

After a total of 24 h from the irradiation, an MTT tetrazolium salt (3-(4,5-dimethylthiazol-2-yl)-2,5-diphenyltetrazolium bromide) test was performed to evaluate cell viability. Here, 100 µL of MTT (2.5 mg/mL) was added to 100 µL of DMEM without phenol red; the solution was added to each well and left in contact with the cells for 3 h in the incubator. After removing the MTT from the wells, 300 µL of dimethyl sulfoxide (DMSO; Sigma-Aldrich, Milan, Italy) were added to lysate the cellular and mitochondrial membranes and solubilize the formazan. Next, 100 µL of each sample were taken and transferred, respectively, to a 96-well plate (Cellstar 96 Well Culture Plate; Greiner Bio-One, G) having an area of 0.28 cm^2^. The absorbance of each well was detected with a spectrophotometric method using an ELISA Plate Reader (iMARK Microplate Absorbance Reader; BioRad, Milan, Italy) at 570 with 690 nm as the reference wavelength. Cell viability was calculated as a percentage ratio of the absorbance of the sample to the absorbance of the control.

### 2.8. Statistical Analysis

The statistical analysis was performed by means of Statgraphics Centurion XVI software (2017 Statgraphics Technologies, Inc., The Plains, VA, USA). Analysis of variance (ANOVA, one way) was followed by Fisher’s least significant difference (LSD) procedure analysis to determine significant differences between samples at the 95% confidence level.

## 3. Results and Discussion

### 3.1. Functionalization of the CS-OA-PLGA NPs with ICG

**Figure 1:**
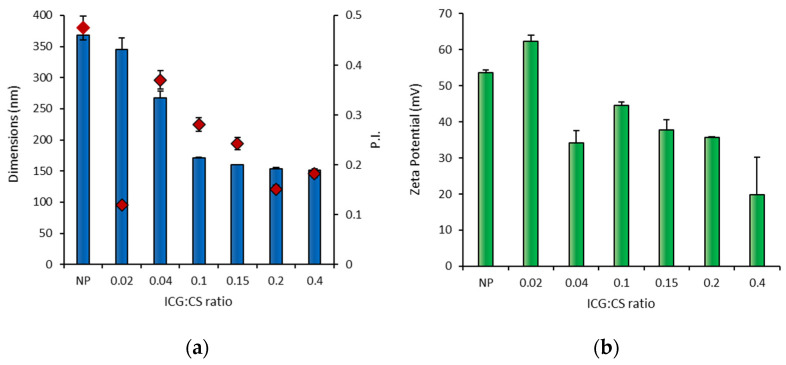
Dimensions, polydispersity index (PI red diamond symbols) (**a**) and zeta potential values (**b**) of unloaded NPs without ICG (NP) and after addition of increasing ICG up to an ICG:CS ratio of 0.4 (mean values *n* = 3 ± s.d.).

Figure 1a shows the dimensions of the CS-OA-PLGA NPs that were here functionalized with ICG by adsorption. In Figure 1b, the corresponding variation of zeta potential is reported. ICG was added at increasing molar ratios with respect to the CS used as CS-OA at a constant amount to stabilize the NPs. Due to the acidic nature of ICG, an ionic interaction occurs at the surface of the NPs. It was previously demonstrated that a layer of CS-OA was arranged at the droplet surface during the emulsion step. Thanks to the amphiphilic behaviour of the modified polysaccharide, this layer endures on the final PLGA NPs surface, generating a positive zeta potential [37]. The dimensions, about 350 nm, and the strongly positive zeta potential of NPs before the addition of ICG (sample NP in Figure 1a,b) confirmed previous findings [37]. Aside from that, the decrease in zeta potential after the addition of an increasing amount of ICG, which led to a partial neutralization of the CS charges, revealed a conceivable interaction between ICG and the CS shell surrounding the NPs. All the samples obtained by increasing the amount of ICG added up to a molar ratio of 0.4 show dimensions of a few hundred nm. By adding ICG until a 0.1 molar ratio was obtained compared with the amount of CS-OA, a clear decrease in dimensions was observed, followed by a stabilization of the dimensions that remained unchanged for further ICG addition. In all cases, the PI was below 0.5, corresponding to an appropriate sample uniformity. The data suggest a stabilizing effect of the dye on the NP dimensions. To proceed with the study, the ICG:CS ratio of 0.4:1 was chosen to prepare all the functionalized NPs, both by adsorption and by encapsulation. 

**Figure 2:**
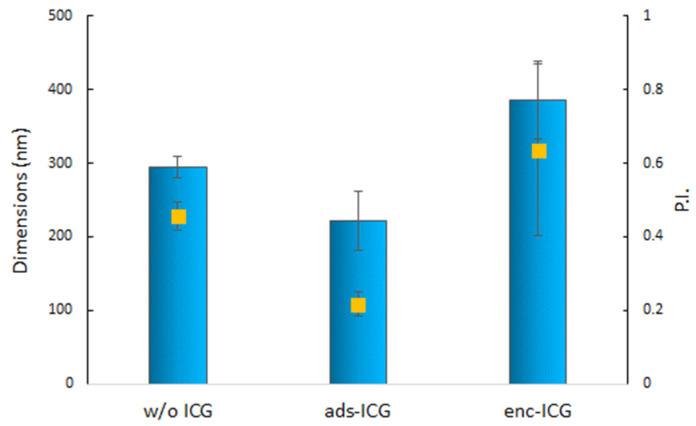
Dimensions and polydispersion index (PI square yellow symbols) of RSV-loaded NPs without ICG (w/o ICG) and with ICG associated by adsorption (ads-ICG) and by encapsulation (enc-ICG) (mean values *n* = 3 ± s.d.).

In Figure 2, the dimensions of the NPs functionalized with ICG both by adsorption and by encapsulation methods at the selected 0.4:1 molar ratio and loaded with RSV are reported. The dimensions of the RSV-loaded samples (w/o ICG) were in accordance with those of similar NPs previously described by Miele et al. [41]. The addition of ICG by adsorption (ads-ICG) led to a slight reduction in dimensions, confirming the stabilizing effect observed in Figure 1a. The quite low PI value, in this case, was around 0.2, indicating a narrow dimensional distribution. On the contrary, enc-ICG NPs showed larger dimensions in comparison with the unloaded sample, up to 400 nm, and a less homogeneous dimensional distribution, indicated by a larger PI value. Even in the case of RSV-loaded NPs, the functionalization method led to a zeta potential decrease from about 50 mV to 10.2 ± 1.3 mV in the case of ads-ICG NPs and to 20.2 ± 2.0 mV in the case of enc-ICG NPs. This result could suggest that part of the ICG added during the preparation of enc-ICG NPs remained at the NP surface. Further characterization has been performed for comparison purposes on the NPs obtained with both the association methods. 

### 3.2. UV-Visible and Fluorescence Spectroscopy

**Figure 3:**
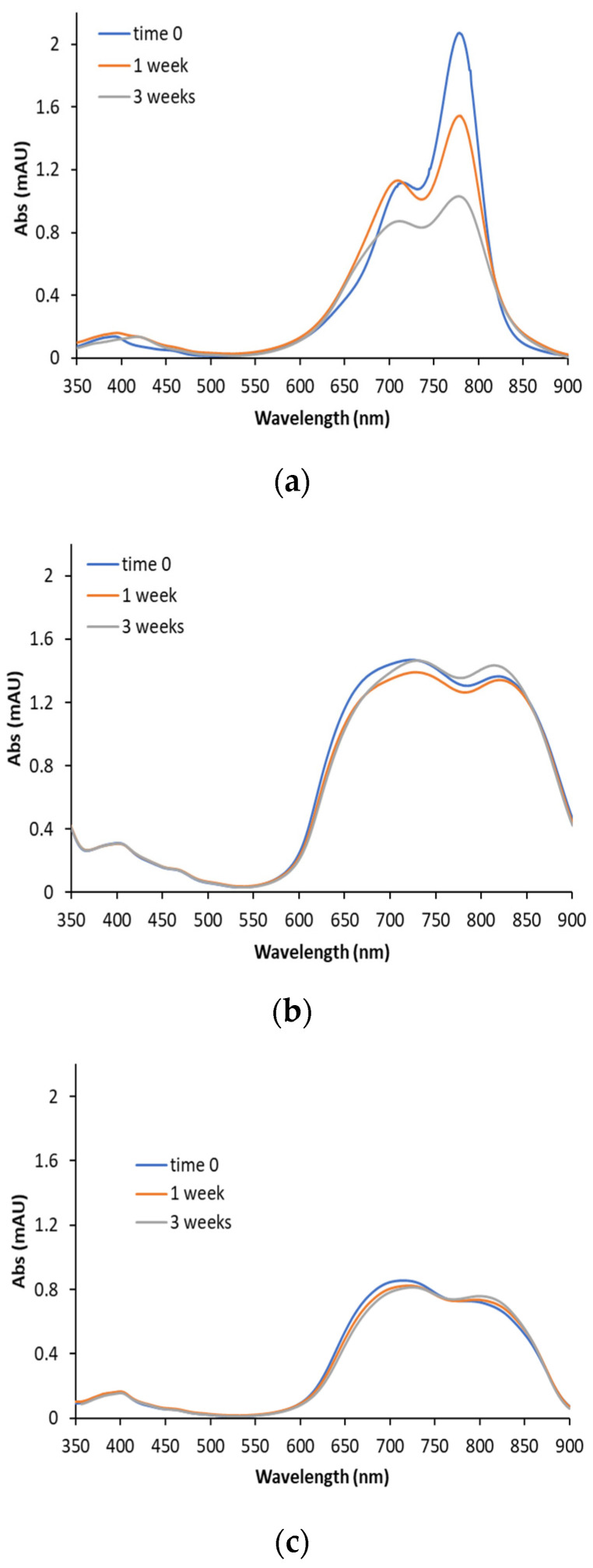
UV-Vis spectra registered at the preparation (time zero) and after 1 and 3 weeks at 4 °C for ICG in aqueous solution (**a**) and for ads-ICG NPs (**b**) and enc-ICG NPs (**c**).

It is well known from the literature that ICG aqueous solutions are characterized by low stability [31]. This is confirmed by the changes reported in Figure 3a in the UV-visible spectra of a 0.01 mg/mL solution of ICG in distilled water at time zero and after 1 and 3 weeks of storage at 4 °C. After 1 week, the spectrum was already modified, with a bathochromic effect evident by the shift of the main peak towards longer wavelengths. Moreover, a modification of the absorption ratio between the two peaks, at about 710 nm and 783 nm, was evident. Both these effects were more pronounced after 3 weeks, indicating the intrinsic molecule instability.

The UV spectra of Figure 3b,c were acquired at time zero and after 1 and 3 weeks by evaluating the NPs with ICG associated by the adsorption (3b) and by encapsulation (3c) methods. Compared with the ICG in solution, the spectrum of Figure 3b appeared different. Although the absorption is still mainly in the 600–900 nm region, a strong variation in shape was appreciable, in accordance with the literature findings for samples obtained by the interaction of ICG with CS [31]. This supports the hypothesis of the generation of an ICG-CS complex caused by ionic interaction between the anionic ICG molecule and the amino groups of the CS-OA placed on the surface of the NPs. The maximum absorbance of ICG shifted to 715 nm and 810 nm in both ads-ICG NPs and enc-ICG NPs, and the intensity ratio provided different results compared to ICG in an aqueous solution. However, the modification of the spectrum by the time during the evaluation time frame was less marked. 

In enc-ICG NPs (Figure 3c), further differences compared to ICG solution and even to ads-ICG NPs were displayed. The two peaks were revealed at 730 and 830 nm, confirming a stronger shift, while the intensities, such as in ads-ICG, were comparable. In accordance with what was observed with ads-ICG NPs, relatively low modifications were induced by the 3 weeks of storage.

**Figure 4:**
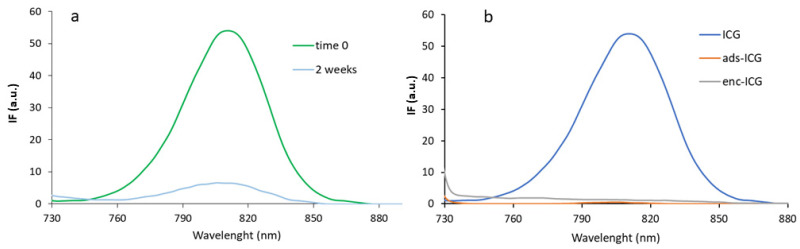
Fluorescence spectra of ICG in aqueous solution at time zero and after 2 weeks at 4 °C (**a**), and of ICG in aqueous solution (ICG, repeated for comparison) and associated with NPs (ads-ICG and enc-ICG) at time zero (**b**).

Figure 4a shows fluorescence spectra of an ICG solution in distilled water compared with the same solution after 2 weeks of storage at 4 °C. An intense emission peak characteristic of the molecule was evident at 820 nm wavelength, and a reduction of intensity due to time instability, in line with the UV-Vis results, was recorded. In Figure 4b, the same ICG spectrum is compared with the spectra obtained with ads-ICG NPs and with enc-ICG NPs. In both the NP samples, the emission of ICG was not visible anymore. For ads-ICG NPs, this behavior indicates a quenching phenomenon conceivably due to the ionic interaction of the dye with the amino groups of CS at the surface of the NPs. A similar strong quenching effect is, however, also visible for the enc-ICG NP sample.

**Figure 5:**
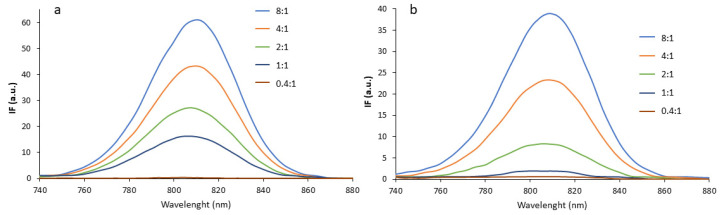
Fluorescence spectra of ICG after interaction with NPs (**a**) and with CS (**b**) at different ICG:CS molar ratios.

To better clarify the effect of the ionic interaction between ICG and CS, a solution of ICG was put in contact with increasing concentrations of NPs. The results are illustrated in Figure 5a, where it is possible to see that the fluorescence decreased with the increase in the amount of CS-coated NPs added, from 8:1 to 0.4:1 ICG:CS molar ratio. The signal was completely quenched for a ratio of 0.4:1, confirming the results reported in Figure 4b. In Figure 5b, analogous results are shown after the addition to the same ICG solution of different amounts of CS-OA polymer at the same ICG:CS molar ratios. A quenching depending on the ICG:CS molar ratios was observed, although, comparing the two figures, it seemed that, at every theoretical ratio, a lower residual fluorescence intensity remained after interaction with free CS-OA polymer than with NPs. This can be explained by the hypothesis that the free polymer is more available for interaction with the dye, while the more rigid conformation of CS-OA at the NP surface reduces the availability of CS amino groups for interaction with ICG.

### 3.3. FTIR Characterization

**Figure 6:**
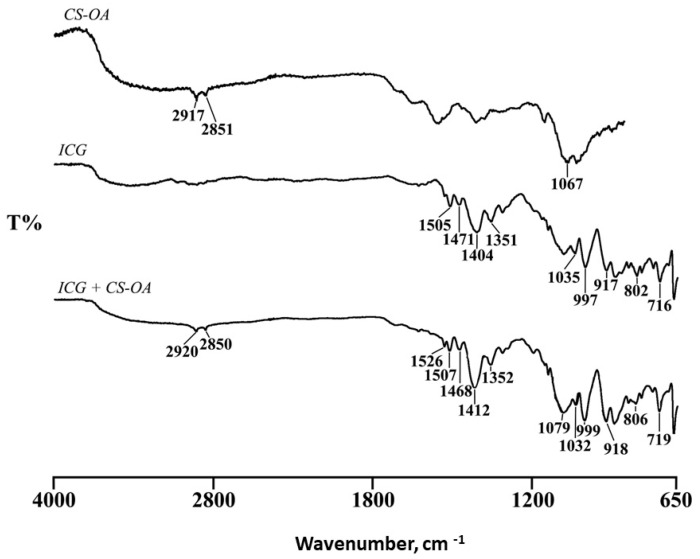
FTIR spectra of the CS-OA, ICG and their interaction product at a 0.2:1 molar ratio.

To highlight possible chemical modifications due to ICG and CS-OA ionic interaction, FTIR spectra were collected for CS-OA, ICG, and for their interaction product at a 0.2:1 ICG:CS molar ratio, for which no excess of ICG was supposed (Figure 6). 

The CS-OA spectrum showed two bands at 2917 and 2851 cm^−1^ due to C-H stretching and the characteristic C=O stretching band at 1711 cm^−1^. These bands, even if of lower intensity, were still present in the FT-IR spectrum of the interaction product, confirming the presence of CS-OA, together with the ICG characteristic bands at 1507, 1471, and 1412 cm^−1^, in the CH_3_ asymmetric bending and C-N stretching regions.

The shift of ICG bands to higher wavenumber in the ICG-CS-OA spectrum compared to that of ICG alone confirmed an ionic interaction between the two components.

### 3.4. Fluorescence Behavior in Cell Culture Medium

**Figure 7:**
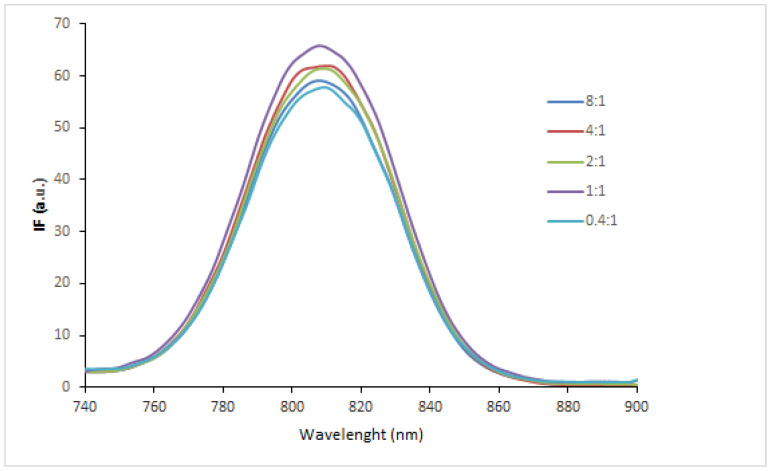
Fluorescence spectra of ICG after interaction with NPs at different ICG:CS molar ratios in cell culture medium.

In the perspective of the evaluation of the systems in cell cultures, the fluorescence spectra of the same ICG NP ratios considered in Figure 5a were also investigated by diluting the samples in the DMEM cell medium without phenol red. The fluorescence spectrum of ICG dissolved in the medium was similar to that of ICG dissolved in distilled water (Figure 4a). The ICG spectra acquired after mixing CS-OA NPs at different molar ratios to ICG dissolved in cell medium are illustrated in Figure 7. The results revealed that the fluorescence was not modified by the addition of CS-coated NPs, suggesting that the interaction between ICG and CS is reversible after dilution in the medium. This evidence can be partially related to the pH of the medium, which is above the pK_a_ of CS [31], and partially to the presence in the medium of the serum proteins that compete with CS for the interaction with ICG. It is well known, in fact, that the ICG easily interacts with proteins such as albumin and transferrin, probably both by electrostatic and hydrophobic interactions [45,46]. 

**Figure 8:**
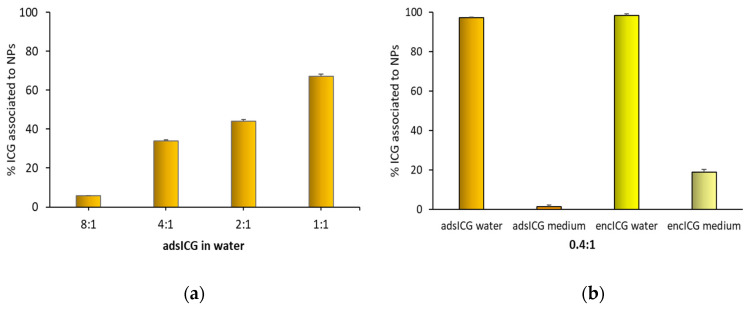
ICG associated with NPs. (**a**) Effect of different ICG:CS ratios for ICG loaded by adsorption in distilled water. (**b**) Effect of adsorption or encapsulation loading methods in distilled water or in DMEM medium (medium) for ads-ICG NPs and enc-ICG NPs at a 0.4:1 ICG:CS molar ratio.

To better focus on these aspects, free ICG was separated from IGC associated with the NPs by using a Sephadex G-25 column. Dialysis separation was not successful because a significant quote of ICG was adsorbed onto the cellulose membrane of dialysis bags. Preliminary experiments showed that ICG was completely eluted from the Sephedex column used by 12 mL of elution volume. ICG associated with the NPs was instead completely retained in the column. Figure 8a reported for ads-ICG NPs the results of different elution experiments performed in distilled water with samples with different ICG:CS ratios. It is possible to see that the percentage of ICG not eluted from the column and therefore associated with the NPs increased with the increase in the amount of NPs mixed with ICG before their loading in the column. This result puts in evidence the saturation level of ICG loading on the NPs by the adsorption method. About 70% of ICG was bound to NPs when the ICG:CS molar ratio was 1:1, and this percentage increased to about 100% with the 0.4:1 ratio, as reported in Figure 8b.

At the ICG:CS molar ratio of 0.4:1 a comparison was performed between ads-ICG and enc-ICG NPs. In Figure 8b, the differences in elution behavior of these samples are compared in experiments performed either in distilled water or in the cell culture medium. In distilled water, the behavior of the two samples was superimposable, and all the ICG was associated with the NPs. In the elution experiments performed in the medium, in the case of the ads-ICG NPs, the association of ICG and CS in the medium was almost zero, confirming what was previously observed (Figure 7). The medium could fully displace ICG from the surface of the NPs. On the other hand, for enc-ICG NPs samples, with ICG loaded in a PLGA core, in the medium, about 20% of the ICG was still associated with the NPs. This percentage can be assumed as the amount of ICG effectively encapsulated inside the NPs, giving a quantification of the encapsulation efficiency (EE) of ICG for this kind of NPs. The remaining 80% was probably not encapsulated and ionically associated with the CS at the NP surface during the NPs preparation. The quite low percentage of ICG encapsulated in the PLGA NPs core can be explained by the hydrophilic character of the molecule. 

Loading of ICG by adsorption is quite commonly described in the literature for NPs based on albumin, whose easy interaction with ICG is exploited to improve the dye fluorescence intensity and its distribution in the lymphatic system [47] and to load it in HSA NPs with improved PDT and PTT effect [20]. Considering CS, ionic complexes between ICG and an arginine derivative of CS activate the formation of self-assembled NPs, as described by Jheng et al. [31]. Less common is the surface adsorption of ICG on previously prepared NPs, an approach quite widely used for the coating of NPs with polymers and sometimes proposed with small molecules, such as, for example, folic acid [48].

Despite the labile interaction between ICG and the CS amino groups at the NP surface, the adsorption approach presents some advantages. The loading of ICG occurs, in fact, quickly, in a very easy way, and leads to a relatively high amount of ICG associated with the NPs in comparison with the encapsulation method, as demonstrated by the results obtained here. The amount loaded by adsorption was about five times higher than that loaded by encapsulation in the NP core. The adsorption approach also makes the association of ICG with drugs in NPs easier, such as in this case with resveratrol, selected as a model molecule. In the present case, the EE was, in fact, relatively high for the more hydrophobic RSV, corresponding to 78.6% ± 8.4 as a mean of the batches prepared in the present work, confirming the value previously obtained [37], while the EE was around 20% for the more hydrophilic ICG. This scenario can be even worse in the case that it would be necessary to encapsulate highly hydrophobic drugs that could require the design of an NP core by choosing even more hydrophobic polymers and apolar solvents for the preparation procedure. This could involve an even more difficult encapsulation of ICG inside the NP core. The possibility of interaction between the encapsulated drug and the dye should be moreover carefully verified time by time in the case of their co-localization in the NP core, while lower compatibility concerns should arise in the case that the drug is localized in the NP core and the dye around the NP shell. A further advantage of the adsorption method involves the combination of NPs with ICG solutions extemporarily prepared, therefore reducing stability concerns for the dye.

### 3.5. Caco-2 Cells Interaction

**Figure 9:**
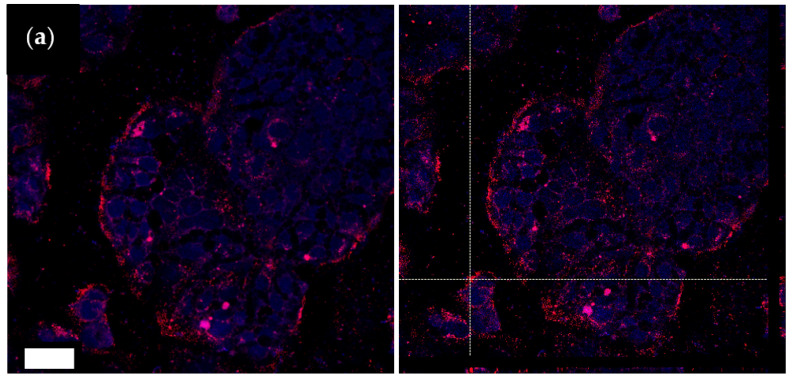

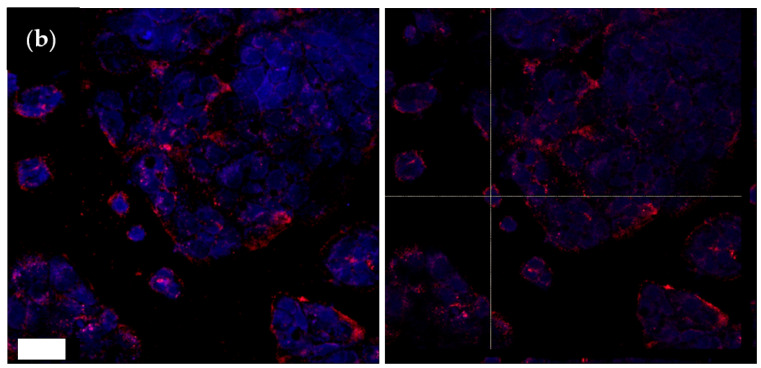
CLSM image (**left**) and z-projections (**right**) of Caco-2 cells after 24 h exposure to Nile red loaded NPs not functionalized (**a**) and functionalized with ICG (**b**) by adsorption at 0.4:1 ICG:CS ratio (ads-ICG NPs). Bar = 50 µm.

In Figure 9, a CLSM image illustrates the interaction of the ICG-coated NPs with the Caco-2 cells. The NPs, loaded with Nile red as a tracer, are visualized in red. The confocal analysis revealed that the NPs were internalized in the cells close to the nuclei (stained in blue). The positive internalization of NPs coated with CS-OA, here assessed for comparison, can be explained by the well-known positive effect of CS positive charge [41]. In the case of the sample functionalized with ICG, the results suggested that the layering of ICG around the NPs did not impair their affinity for the cells and, therefore, the internalization, despite the shielding of the CS shell and the reduction in the zeta potential. It must be considered that, according to some literature evidence, ICG itself is capable of interacting with membrane phospholipids, triggering endocytosis mediated by clathrin [6]. This mechanism could potentially contribute to cell internalization when ICG is present at the NP surface, such as in the present case.

**Figure 10:**
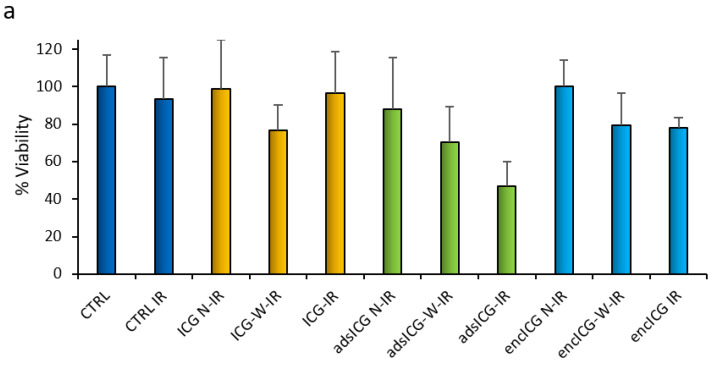

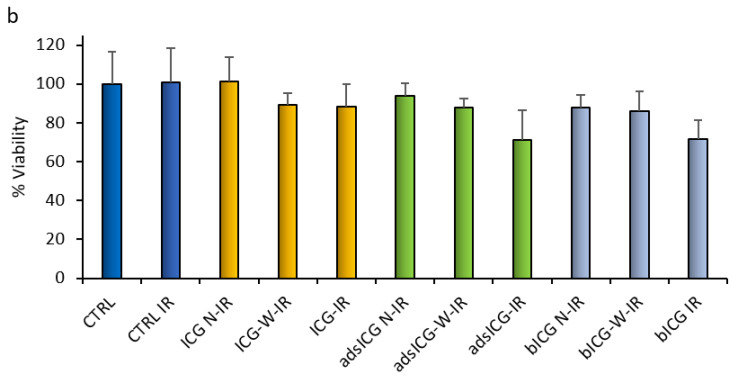
Caco-2 cell viability percentage (with respect to the controls) after 24 h exposure to the samples. Samples were not irradiated (N-IR) or irradiated after sample washing (W-IR) or without washing (IR) for 15 min at 0.1 W/cm^2^ (**a**) or 5 min at 0.3 W/cm^2^ (**b**). CTRL: untreated cells (controls). IR: irradiated samples. N-IR: samples not irradiated. W-IR: cells maintained in contact with the samples for 24 h and washed just before irradiation. ICG: dye solution. adsICG: ICG loaded NPs by adsorption. encICG: ICG-loaded NPs by encapsulation. bICG: NPs without resveratrol, ICG-loaded by adsorption. Mean ± s.d., *n* = 8.

Figure 10a,b shows the results of Caco-2 cell viability after 24 h exposure to ICG solution and after irradiation with a laser beam at 803 nm, either 100 mW/cm^2^ for 15 min (Figure 10a) or 300 mW/cm^2^ for 5 min (Figure 10b). Irradiation was performed on cell substrates treated in the presence of the samples (at the end of 24 h exposure) and after removing it by washing just before irradiation (W). In both cases, ICG solution and ads-ICG NPs were compared with cells not treated and not exposed to radiation (CTRL) or cells not treated but irradiated using the same operating parameters (CTRL IR). In Figure 10a, the ads-ICG NPs were also compared with the enc-ICG NPs. In all cases, the concentration of ICG was kept at 13 µM while, considering the EE%, the RSV concentration was 80 µM. The quite low concentration of ICG was possibly responsible for the absence of the aggregation phenomenon, which was instead observed in the literature for concentrations higher than 60 µg/mL [31]. A batch of NPs without RSV, loaded with ICG by adsorption (bICG sample), was considered for comparison (Figure 10b).

The trend observed for ICG solution and ICG adsorbed on NPs was quite similar for the two different irradiation conditions. Considering the ICG solution, no statistically significant decrease in cell viability was observed between the control (CTRL) and the cells exposed to ICG without irradiation (ICG N-IR), while a slight but statistically significant difference in viability occurred between the controls irradiated (CTRL IR) and free ICG irradiated in the cases in which ICG solution was removed before irradiation (ICG-W-IR). For ICG not washed away, this effect was less clear and more variable. This suggests that the ICG internalized (ICG-W-IR) reduced the cell viability by irradiation, as expected, but ICG still present in solution in the wells (ICG-IR) absorbed the energy of the laser beam without causing cell damage.

The viability of Caco-2 cells treated with the NPs systems with ICG adsorbed (ads-ICG) decreased more after irradiation than without the irradiation step. In fact, the CTRL vs. ads-ICG N-IR comparison was statistically not significant for both the irradiation conditions (Figure 10a,b), indicating a lack of toxicity of the NPs if not irradiated. For the same sample, when washing was not performed, the irradiation was more effective in reducing viability than for the cells treated but washed with PBS before irradiation. In fact, ads-ICG-W-IR and ads-ICG-IR were both statistically different (*p* < 0.05) from CTRL-IR for each experimental set (a and b), and in both cases, the viability for ads-ICG-IR was statistically lower than that of ads-ICG-W-IR (*p* < 0.05). This evidence suggests that the presence of NPs in the culture medium supported and extended the PDT effect of the internalized NPs. It is moreover clear that the PDT effect was higher and more effective by treating the cells with ads-ICG NPs than using free ICG, as viability was significantly lower for ads-ICG-IR than for both ICG-IR and ICG-W-IR (*p* < 0.05). A role in this result may be played by the ability of the ads-ICG NPs to concentrate ICG around them, locally increasing the efficiency of its PDT effect. The direct comparison between RSV-loaded and -unloaded samples did not show statistically significant differences, as shown in Figure 10b, where for the unloaded NPs functionalized with adsorbed ICG (bICG IR), a significant effect, attributable to the adsorbed ICG, was observed after irradiation, as bICG IR was significantly lower than CTRL IR (*p* < 0.05), but not different from ads-ICG IR. These results suggest that no synergy can be here observed between RSV loaded in NPs and adsorbed ICG. 

Even in the case of enc-ICG NPs, the irradiation affected the cell viability, leading to a reduction in surviving cells, significant (*p* < 0.05) for both enc-ICG-W-IR and enc-ICG-IR compared with enc-ICG N-IR. Again, when ICG was associated with NPs, the irradiation always produced a statistically significant reduction in cell viability compared with the irradiated ICG solution. An easier internalization of NPs by endocytosis and higher concentrations of ICG associated with NPs, especially but not only after cell uptake, can explain this evidence.

No appreciable improvement of enc-ICG with NPs on cell viability reduction compared with ads-ICG NPs could be found. It is possible to argue that in the case of in vitro assays, considering the small volumes of cell medium, the dilution of the sample was not so high as to induce a competition suitable to displace ICG from the reversible interaction with CS-OA NPs. This could encourage the use of this system for cancer loco-regional therapy, where the dilution due to blood circulation is avoided, and the adsorbed ICG could still remain associated with the NPs. 

As previously pointed out, PDT and PTT involve acute inflammation and induction of the immune response due to the presentation of tumor-derived antigens to T cells. This mechanism has previously been described for PDT [49], and its importance is still recognized in recent literature, where PTT and PDT immunotherapies are outlined [40,50,51]. Cell death by necrosis and photothermal ablation can generate tumor-associated antigens triggering a host immunity that, if based on the PTT mechanism alone, is considered too weak to obtain durable effects. The association of nano-based PDT-PTT with immune adjuvants has recently been considered promising to overcome this limit [51]. 

In this perspective, the association of ICG with NPs coated with CS-OA appears promising, as the NP system showed in PBMCs an increase in cytokines related to inflammatory response and immune response activation, such as interleukin 6 (IL6) and tumor necrosis factor α (TNF-α) [38]. Specific studies will be useful on the ads-ICG-NP system described here, exploring the combination of the immunomodulatory effect of the NPs with ICG irradiation. 

## 4. Conclusions

The results obtained here confirm the NPs coated by CS-OA as a versatile system that can be easily loaded with hydrophobic drugs in the PLGA core, maintaining the CS shell available for easy electrostatic interactions with anionic moieties, like ICG. This interaction appeared reversible in a complex medium like DMEM, although the extent of the dilution was a critical factor, and a clear effect on cell viability after irradiation could be observed for ads-ICG NPs in the cell culture test, confirming the positive role of the interaction between ICG and the NPs. The sensitivity to the dilution, however, suggests the use of the ads-ICG NPs for loco-regional delivery instead of systemic administration, where the higher dilution of the sample, in blood circulation, for example, would result in complete dissociation of ICG from the NP surface. The easy and effective ICG association with CS-OA coated NPs by adsorption opens the opportunity to exploit the combination of the PDT effect of ICG with the properties of CS-OA coated NPs, for which good internalization and immunostimulatory effects were previously demonstrated. This aspect deserves further investigation.

## Data Availability

Data are available from the corresponding author.
